# Inconsistencies in mapping current distribution in transcranial direct current stimulation

**DOI:** 10.3389/fnimg.2022.1069500

**Published:** 2023-01-16

**Authors:** Anita S. Jwa, Jonathan S. Goodman, Gary H. Glover

**Affiliations:** ^1^Stanford University Law School, Stanford, CA, United States; ^2^Program in Biophysics, Stanford School of Medicine, Stanford, CA, United States; ^3^Department of Radiology, Stanford University, Stanford, CA, United States

**Keywords:** transcranial direct current stimulation, transcranial electrical stimulation, non-invasive neuromodulation, current mapping, functional magnetic resonance imaging

## Abstract

**Introduction:**

tDCS is a non-invasive neuromodulation technique that has been widely studied both as a therapy for neuropsychiatric diseases and for cognitive enhancement. However, recent meta-analyses have reported significant inconsistencies amongst tDCS studies. Enhancing empirical understanding of current flow in the brain may help elucidate some of these inconsistencies.

**Methods:**

We investigated tDCS-induced current distribution by injecting a low frequency current waveform in a phantom and *in vivo*. MR phase images were collected during the stimulation and a time-series analysis was used to reconstruct the magnetic field. A current distribution map was derived from the field map using Ampere's law.

**Results:**

The current distribution map in the phantom showed a clear path of current flow between the two electrodes, with more than 75% of the injected current accounted for. However, in brain, the results did evidence a current path between the two target electrodes but only some portion ( 25%) of injected current reached the cortex demonstrating that a significant fraction of the current is bypassing the brain and traveling from one electrode to the other external to the brain, probably due to conductivity differences in brain tissue types. Substantial inter-subject and intra-subject (across consecutive scans) variability in current distribution maps were also observed in human but not in phantom scans.

**Discussions:**

An *in-vivo* current mapping technique proposed in this study demonstrated that much of the injected current in tDCS was not accounted for in human brain and deviated to the edge of the brain. These findings would have ramifications in the use of tDCS as a neuromodulator and may help explain some of the inconsistencies reported in other studies.

## 1. Introduction

Transcranial direct current stimulation (tDCS) is a technique that delivers low-intensity direct current (typically 1–2 mA) to the brain through electrodes attached to the scalp. It is “perhaps one of the simplest ways of focally stimulating the brain” (George and Aston-Jones, 2010) and has been investigated for its potential to alter cortical excitability (Nitsche and Paulus, 2011). Application of direct current on the human brain is not a new idea (Priori, 2003), but since the reappraisal of this technique in 2000, it has been gaining momentum as a promising tool for neuromodulation (Dubljevic et al., 2014). Studies have reported the effects of tDCS on various neuropsychiatric diseases, such as depression, chronic pain, stroke, schizophrenia, and Parkinson's disease (Broeder et al., 2015; Szymkowicz et al., 2016; Pinto et al., 2018; Vaz et al., 2019; Fregni et al., 2021). Research has also suggested that application of tDCS can improve a wide range of cognitive functions, including attention span, working and long-term memory, impulse control, language learning, and mathematical ability within healthy subjects [(Campanella et al., 2018; Ke et al., 2019; Lo et al., 2019; Rivera-Urbina et al., 2019), see also review (Filmer et al., 2014)]. On top of these reports on efficacy, the technique's relatively low safety risks, high affordability, and ease of use make it attractive within and outside of the clinical context (Fitz and Reiner, 2013). Consequently, a substantial increase has arisen in the number of tDCS research studies over the last two decades (Dubljevic et al., 2014).

However, recent meta-analyses have revealed significant inconsistencies amongst tDCS studies (Berryhill et al., 2014; Horvath et al., 2015a,b). Berryhill and Martin (2018) showed that in both healthy and clinical populations, the effects of tDCS on cognitive measures are neither robust nor predictable, especially for single session tDCS. Systematic reviews on the efficacy of tDCS have also reported inconsistent outcomes with respect to a specific disorder [for chronic neuropathic pain (Plow et al., 2012); for depression (Mutz et al., 2018; Brunoni et al., 2019); for Parkinson's disease, Alzheimer's disease, Hemi-spatial Neglect, and Aphasia (Cappon et al., 2016)], cognitive function [for episodic memory (Galli et al., 2019); for working memory (Mancuso et al., 2016); for exercise performance (Machado et al., 2018)], and brain region [for prefrontal cortex (Tremblay et al., 2014); for cerebellum (van Dun et al., 2017)]. Small sample sizes, varying stimulation setups, intra- and inter-subject variability, inaccurate localization of electrodes, and the lack of a reliable sham protocol have been suggested as some possible causes for this inconsistency (Horvath et al., 2014; Li et al., 2015; Bikson et al., 2018; Fonteneau et al., 2019).

One critical issue related to tDCS is the lack of empirical data on the distribution of current flow in the brain. How current flows in the brain is fundamental to understanding tDCS because it is the voltage generated by current in resistive tissues that locally alters action potentials and therefore neural firing rates. Studies have also shown that the injected current can generate a wide array of physiological effects involving nerves in scalp, cranial nerves, blood vessels, and astrocytes (Shin et al., 2020; Arora and Dutta, 2022a). Recently, modeling methods have been used to simulate the current flow under specified electrode montages (Bikson et al., 2012; Kessler et al., 2013; Truong et al., 2013; Galletta et al., 2015; Rahman et al., 2015). The computational models have yielded smooth and well-behaved trajectories of current flow between the reference and target electrodes, and many tDCS studies have adopted them to guide the placement of an electrode over the brain region of interest. They have also been used to understand individual variability in tDCS induced-electric fields combined with anatomical scans, to estimate the amount of current that can theoretically be injected into the skull and reach the cortex based on resistive properties of tissue, and to determine a proper dose of current depending on age differences (Ciechanski et al., 2018; Indahlastari et al., 2021). However, only a few studies have attempted to measure the actual current flow, or current induced magnetic field as a marker of the current flow, in the human brain *in-vivo* (Jog et al., 2016, 2020, 2021; Kasinadhuni et al., 2017; Goksu et al., 2018, 2021).

Previous *in-vivo* measurements of current flow in the brain make use of Ampere's law to infer the underlying current distribution from the magnetic field induced by the injected current (Kasinadhuni et al., 2017; Goksu et al., 2018, 2021). When tDCS is performed within an MRI scanner, the resulting magnetic field perturbations in the head cause local proton off-resonance, which in turn alters the phase of the MRI signal as a function of local current magnitude and current flow direction in the brain region. The phase change scales linearly with the axial (z) component of the magnetic field change; therefore, it can be used to create maps of *B*_*z*_. These maps provide information on the underlying current density responsible for the magnetic field perturbations. The fundamental limitation of this approach is that MRI can only detect changes in the z component of the tDCS-induced magnetic field (i.e., that component parallel to the MRI's main magnetic field). Collecting all three spatial components of the magnetic field requires measuring magnetic field changes in at least two tilted orientations of the head, which can be impractical because of subject discomfort with currently available head coil designs and because of the extended scan time of multiple scans. In practice, therefore, the MRI-based current mapping strategy is often inherently constrained by incomplete information. Previous studies have used predictions from computational modeling to fill in the two missing components (Kasinadhuni et al., 2017; Goksu et al., 2018, 2021), but this method makes comparison of the outcome of computation models and actual experiment data circular.

The present study proposes a technique that enables a highly sensitive mapping of magnetic field and current distribution, though still constrained by incomplete field information. The goal of the present study is to experimentally demonstrate the flow of current in the brain undergoing tDCS using a timeseries phase contrast fMRI method. We hypothesize that the differences in the electrical conductivity of cerebral spinal fluid (CSF), gray matter, and white matter cause substantial deviation of current from a desired cortical-based path between the electrodes. This study also aims to quantify how much current actually penetrates into the brain and hypothesizes that due to the non-invasive nature of tDCS, a significant portion of the applied current in tDCS is shunted by scalp, soft tissue, and skull and will flow external to much of the brain volume. If this is true, it would substantially diminish the neuromodulatory ability of tDCS, which may, in part, contribute to the varying results on the efficacy of tDCS reported in the literature.

## 2. Materials and methods

### 2.1. Subjects

Ten healthy subjects were recruited to participate in the study [3 female; average age 48.4 (age range 23–79)]. Subjects were screened for any neurological/psychiatric disorders and for any contraindications to tDCS or MRI. All subjects provided written informed consent using a protocol approved by the Stanford University Institutional Review Board.

### 2.2. tDCS protocol

#### 2.2.1. Human subject experiment

**Figure 1:**
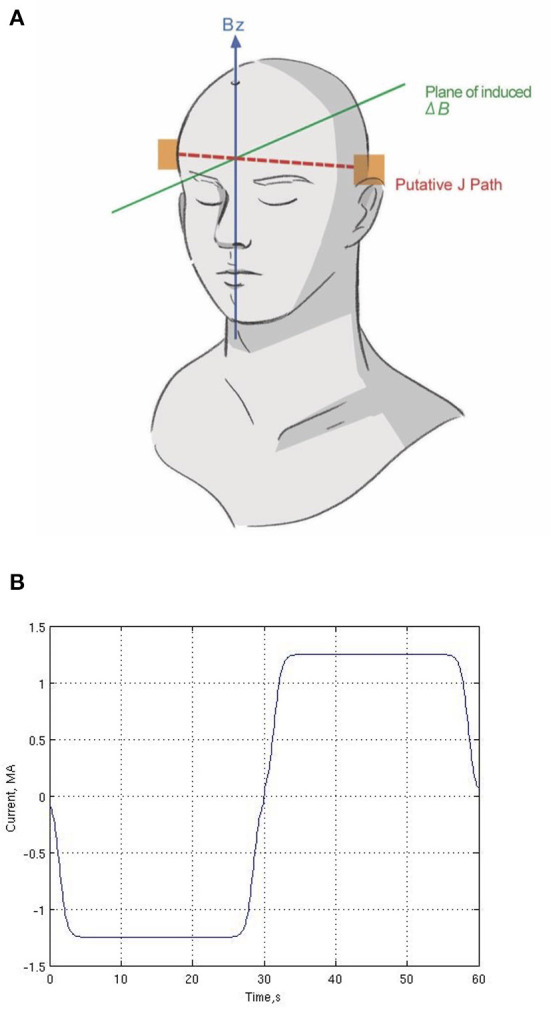
Human subject experiment setup. **(A)** Location of two electrodes (T3 and T4) and putative current (J) path and plane of induced magnetic field (Δ*B*). **(B)** One cycle of the current waveform (repeated six times during the scan). We used a Fermi waveform (±1.25 mA; 60-s period) for 6-min stimulation to maximize the RMS current while controlling for abrupt current transitions.

The electrodes were attached to the scalp at T3 and T4 in the 10-20 EEG System, which are equivalent to T7 and T8 in the 10–10 EEG system (Figure 1A). The electrodes were positioned to maximize the current presumed to flow in a direction perpendicular to *B*_0_ and so that the tDCS-induced magnetic field changes are primarily in parallel with *B*_0_. The electrodes consisted of conductive rubber pads to which wires from the current source were attached and were inserted into saline-soaked sponges (5 × 7 cm^2^). An elastic rubber band was used to securely affix the electrodes to the scalp.

Low-frequency current (±1.25 mA; 60-s period) was delivered for 6 min between the two electrodes in the magnet (Figure 1B). Our protocol is based on principles of block trial design in task-fMRI, which alternates between two states to derive statistical measurements of current flow, not to modulate cognitive function of the brain. By employing a bipolar waveform, the effective current amplitude change is doubled, which provides a robust phase change with which to reconstruct the current flow without increasing the actual current applied, using standard linear statistical signal processing as in fMRI. Thus, reversing the current polarity serves as the “sham” condition, which is zero in direct current. The frequency (60 s period) is too low for reactive current flow effects. While slowly alternating the polarity periodically may induce different neuromodulatory or neurovascular effects than direct current, here we are not trying to induce or observe neuronal or neurovascular changes.

We used Fermi functions in place of ramps for our stimulating waveform, as shown in Figure 1B. The Fermi function *F*(t)is defined as


(1) $$\begin{array}{l} F\left(t\right)=\frac{1}{1+\exp\left(\frac{t-t_{0}}{\tau }\;\right)}, \end{array}$$

where t is time, t_0_ = 1.4 s, τ = 0.5 s. These parameters were chosen after preliminary trials to maximize the RMS duty cycle of current delivered while simultaneously reducing unpleasant tingling and itching under the electrodes that would otherwise result from abrupt changes in current intensity.

Each subject underwent four consecutive scans with stimulation, so the total stimulation time was 24 min. The scans were repeated with no interaction with the participants and with minimal delay between scans. Electrical stimulation was generated by a custom-built bipolar constant current source controlled by a digital-to-analog convertor, with a maximum output voltage range of ± 15 v, which is substantially lower than other commercially available tDCS devices to maximize safety in the magnet (Caputron, 2020). The electrode wires were routed from the source in the control room through the scan room wall with radio frequency (RF) filters to eliminate the injection of noise into the MRI images. A series 1 Kohm resistor together with a 1 mHy RF choke was inserted between each feed wire and its electrode (near the electrode) to limit potential RF current induced by the 128 MHz MRI excitation. After exiting the electrodes, the wires were positioned several centimeters away from the head by non-conductive foam pads and then kept parallel to B_0_ to limit the contamination of the tDCS-induced magnetic field in the head by fields generated by the wires themselves.

#### 2.2.2. Phantom experiment

**Figure 2:**
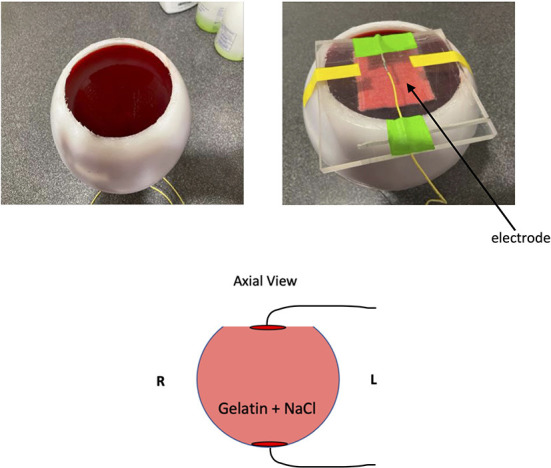
Phantom experiment setup. We crafted a gelatin phantom doped with NaCl to reduce T1 as well as add electrical conductivity. In the cartoon diagram, the two red disks on the top and bottom of the phantom indicate the location of electrodes.

To validate our method of reconstructing current flow with only the *B*_*z*_ component of measured magnetic field, we utilized a phantom that has more uniform properties than a human brain does. A 17 cm diameter hollow spherical plastic phantom (Dielectric Corp, Madison, WI) was filled with commercially available gelatin (Jell-O., 2022), which was was doped with NaCl to approximate the average conductivity of human brain tissue (~0.5 S/m). About 1 cm of the top was removed to allow filling the shell. The current was delivered through an aluminum foil electrode (5 × 7 cm^2^) on the bottom of the phantom and a conductive rubber electrode inserted into saline-soaked sponge (5 × 7 cm^2^) resting firmly on the surface of the gelatin on top of the phantom (Figure 2). The stimulation protocol and the top electrode were identical to those in the human subject experiments, as was the analysis. The bottom electrode was of aluminum to facilitate fabrication of the phantom. Note that the geometry of the phantom experiment has presumed direction of current flow predominantly vertical instead of predominantly horizontal as in the human subject experiments.

### 2.3. MRI data acquisition

fMRI data were collected using a 3T scanner with a 48-channel head coil (GE Premier, Milwaukee, WI). Twenty-four oblique axial slices were acquired with 5 mm slice thickness with 0 mm skip. T2-weighted FSE structural images (TR = 3,000 ms, TE = 68 ms, ETL = 12, FOV = 22 cm, matrix = 256 × 192) were collected for anatomical reference. Phase contrast timeseries images were acquired during 6-min scans to obtain the *B*_*z*_ component of the magnetic field induced by the time-varying injected current. A gradient echo spiral-in/out pulse sequence was employed (Glover and Law, 2001) (TR = 1,500 ms, TE = 30 ms, flip angle = 70°, FOV = 22 cm, matrix = 64 × 64, 3.4375 × 3.4375 mm in-plane resolution, 240 time frames, same slice prescription as the anatomical volume). The spiral-in/out sequence recovers signal in frontal-orbital regions normally lost in EPI acquisitions due to off-resonance induced by nasal air cavities (Glover and Thomason, 2004; Glover, 2012). Phase contrast maps were reconstructed using tSNR weighting to combine the spiral-in and spiral-out images. Three initial time frames (volumes) were discarded to allow for T1 equilibration, and the second retained phase volume was subtracted from all volumes to eliminate constant phase offsets and obviate the need for phase unwrapping. Second order phase drifts were eliminated by voxel-wise detrending across time frames. Δ*B*_*z*_*(t)* maps were calculated from the resulting measured phase ϕ_*m*_ *(t)* using


(2) $$\begin{array}{l} \Phi_{m}\left(t\right)=\gamma \Delta B_{z}\left(t\right)\;TE, \end{array}$$

where γ is the gyromagnetic ratio of protons. In the phantom experiment, anatomy and phase contrast images used acquisition parameters identical to those of the human experiments.

No heating and no image artifacts were observed in preliminary tests during scanning with a phantom. The measured magnetic field in the head could have contributions from extra-corporal fields generated by the electrode wires since the wires have the full current flowing within. These contributions would distort the *B*_*z*_ measurements. To test this, the mapping experiment was also conducted on a cantaloupe phantom with a larger single-channel birdcage head coil with the wires positioned in the configuration normally used as well as with the wires run parallel to the B_0_ axis directly from the electrode in the superior direction (top of the phantom). By placing the wires as close as possible to the phantom but then leading them in the opposite direction to the normal configuration, this experiment maximizes the possibility of observing a difference in distortion of the magnetic field in the phantom. The *B*_z_ maps did not differ significantly from the maps obtained by the normal configuration, suggesting that the electrode wires did not substantially distort the measurements.

In addition, we further tested the sensitivity of our measure of *B*_z_ fields. We generated the *B*_z_ field maps in a NaCl-doped water phantom at different current amplitude (1.25 mA to 0 m, with 0.25 decrement) (Supplementary Figure 1A). The beta values of an ROI in positive magnetic field in the *B*_z_ maps showed linear decrease as the current amplitude decreases (Supplementary Figure 1B).

### 2.4. MRI data analysis

A standard fMRI general linear model (GLM) processing pipeline (Worsley and Friston, 1995) was employed to obtain magnetic field maps from the timeseries phase maps. Preprocessing included slice-timing correction and spatial smoothing with a 5 mm isotropic Gaussian filter. No mask was employed at the edge of the brain to avoid artificial abrupt discontinuities. The GLM correlated timeseries phase data with the injected current waveform shown in Figure 1B to generate maps of beta values (proportionality between measured magnetic field and the model waveform) of the signed field Ampere's law was then used to calculate the current densities in a plane perpendicular to z; as noted earlier, we only have partial information, namely ∂*B*_z_/∂*y* and ∂*B*_*z*_*/*∂*x* (Kasinadhuni et al., 2017), so the currents are denoted $\tilde{J_{x}},\;\tilde{J_{y}}$:


(3) $$\begin{array}{l} \mu_{0}\;\tilde{J_{x}}=\frac{\partial B_{z}}{\partial y}\;\mu_{0}\;\tilde{J_{y}}=\frac{\partial B_{z}}{\partial x} \end{array}$$

where μ_0_ is the magnetic permeability. This provided current density maps in the xy-plane (i.e., the axial plane, perpendicular to *B*_0_). As noted earlier, the electrode montage was designed to presumably maximize the current flow in the xy-plane. From the J_x_ and J_y_ components in Equation 3 multiplied by the area of the voxel (pixel size × slice thickness), we generated the magnitude and direction of current. Then, we generated an average map of the four consecutive stimulation scans in each subject. We quantified the magnitude of current passing through a 3 pixel-thick vertical (sagittal) plane as a function of Right (R) to Left (L) location of the plane, and tabulated the peak current observed as a function of right-left distance *x*. Finally, these individual subject's maps were normalized into a common atlas (MNI152_T1_2 mm) using the FSL Software Library (https://fsl.fmrib.ox.ac.uk/fsl/fslwiki) and averaged over the 10 subject scans.

Current density maps for the phantom experiment were generated through identical calculation and averaged across the four consecutive scans. The magnitude of the current was quantified by measuring current passing through a 3 pixel-thick horizontal (coronal) plane as a function of Posterior (P) to Anterior (A) location of the plane, because of the 90 degree rotation of the current flow relative to the human subject experiment.

Both for the human subject and phantom experiments, the current injected at one electrode must be fully collected at the other electrode, so the total sum of the current flowing through the test planes is expected to be 1.25 mA. However, the *B*_*z*_ map, and therefore the measured current, is only available within the brain or phantom because there is no MR signal outside. Nevertheless, current-induced magnetic field exists outside the brain but is unmeasured within a few pixels from the brain's edge. Thus, if some portion of the current flows at the brain's periphery through the subdural CSF surrounding the brain, the current accounted for in the maps will be less than that injected.

Finally, to test the repeatability of the derived current distribution maps, differences between the maps across four consecutive scans in the human subject experiment and the phantom were quantified using structural similarity index metrics (SSIM). SSIM compares two images for similarity in luminance, contrast and spatial correlation in local regions of pixel intensities (Zhou et al., 2004). The SSIM values range between 0 (no match) to 1 (perfect match). For both the phantom and each human subject, we took the current distribution map from the first of the four consecutive scans as reference and calculated SSIM between the maps from the reference and second (SSIM12), the reference and third (SSIM13), and the reference and fourth scans (SSIM14) to quantify the changes in the maps. For comparison, we also calculated SSIM between the amplitude maps from the four scans in each human subject as well as the phantom.

## 3. Results

### 3.1. Measurement stability

In both phantom and human studies, the typical measured RMS noise in the phase maps was ~0.75 degrees, resulting in field map RMS noise of ~1.6 nT (Equation 2). This sensitivity demonstrates the value of fMRI-like time series analysis using a GLM in discriminating true stimulation signal from noise caused by physiological processes such as head motion, and cardiac and respiratory pulsation.

### 3.2. Phantom experiment

**Figure 3:**
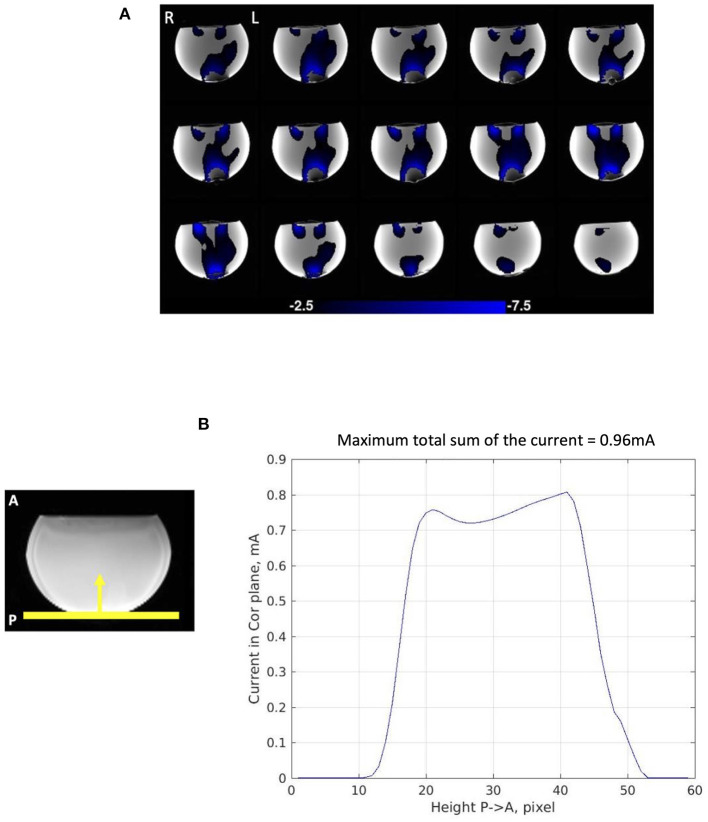
Current distribution in phantom experiment. **(A)** Average current distribution map across the four consecutive stimulation scans showing 3-pixel-thick slab in which total current was measured as slab location was varied. The maps demonstrate Posterior (P) to Anterior (A) current flow (J_y_) in the phantom. **(B)** Magnitude of Posterior (P) to Anterior (A) current flow (J_y_) in the 3-pixel-thick slabs in the coronal plane averaged across the four consecutive scans. The maximum total sum of the current passing through the coronal plane is 0.96 mA.

The phantom experiment demonstrated the validity of our method in mapping current density using time-series phase contrast MR imaging. The average current distribution map of the four consecutive stimulation scans showed almost perfect agreement with the expected current flow between the two electrodes (Figure 3A). The magnitude of upward current (J_y_) passing through 3 pixel-thick slices in the coronal plane from Posterior (P) to Anterior (A) exhibited a clear and consistent current path across the phantom between the two electrodes (Figure 3B). The maximum total sum of the current in the coronal plane was 0.96 mA. This demonstrated that our electrode montage allowed us to capture 76.8% of the injected current (1.25 mA) despite that we used only one (*B*_*z*_) component of magnetic field to calculate current distribution and the magnetic field outside the phantom could not be measured.

The current distributions maps were highly repeatable over the four consecutive scans (**Figure 5A**). The SSIM measures also evidenced the repeatability of the maps with a sightly decreasing trend; 0.903 between the first and the second scan, 0.891 between the first and third scan, and 0.879 between the first and the fourth scan (average 0.891) (**Figure 6A**). The SSIM measures of the amplitude maps were also high and constant across the four consecutive scans; 0.990 between the first and the second scan, 0.988 between the first and third scan, and 0.989 between the first and the fourth scan (average 0.989) (Figure 6B).

### 3.3. Human subject experiment

**Figure 4:**
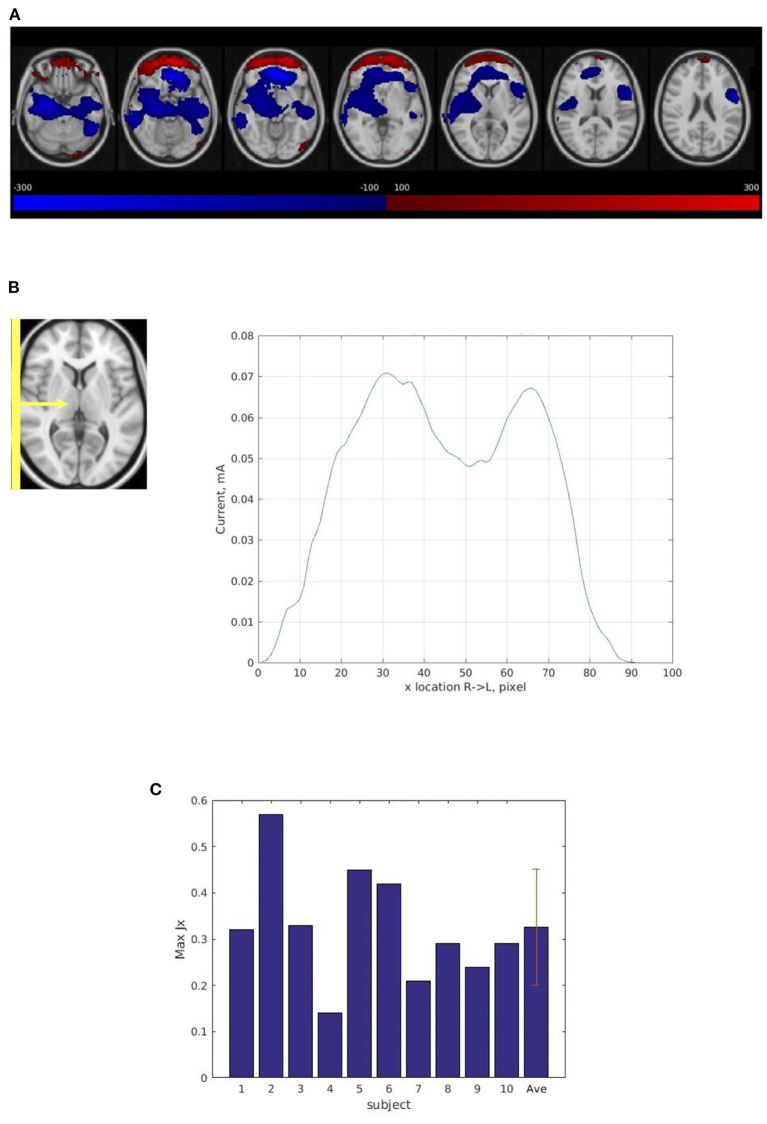
Current distribution in human subject experiments. **(A)** The average current distribution map across ten subjects normalized into a common atlas (MNI152_T1_2 mm) using the T2 anatomic images. The maps show Right (R) to Left (L) (J_x_) current flow in the brain. The blue area shows Right to Left current flow (the primary direction). The red area shows Left to Right current flow which indicates an artifact of boundary conditions at the edge of the brain near the frontal orbital region where susceptibility changes rapidly. **(B)** Magnitude of Right (R) to Left (L) current flow (J_x_) in 3-pixel-thick slabs in the sagittal plane in normalized space averaged across ten subjects' average current distribution maps from the four consecutive scans. The maximum total sum of the current (J_x_) passing through the sagittal plane is 0.07 mA. **(C)** The maximum total sum of magnitude of Right (R) to Left (L) (Jx) current in 3-pixel-thick slabs in the sagittal plane in ten subjects, subject average, and standard deviation. The average maximum total sum of the current passing through the sagittal plane is 0.33 ± 0.12 mA.

The average current distribution map across the ten subject scans confirmed a path between the right and left electrodes (Figure 4A). Some of the current is calculated to be flowing counter to the supposed direction for the montage (e.g., the red-plotted current components in Figure 4A), suggesting an artifact of boundary conditions at the edge of the brain near the frontal orbital region where susceptibility changes rapidly.

However, compared with the results from the phantom experiment, the current path was less evenly distributed in the sagittal plane (Figure 4B) and the average maximum total sum of the right-left current (0.33 mA) was much less than the injected current (1.25 mA) (Figure 4C). The result also showed large variability among the ten subject scans. The average maximum total sum of current in normalized space was substantially lower (0.07 mA) (Figure 4B) than the average total sum of current in subject space (0.33 mA) (Figure 4C) due to this large inter-subject variability (see Supplementary Figure 2).

**Figure 5:**
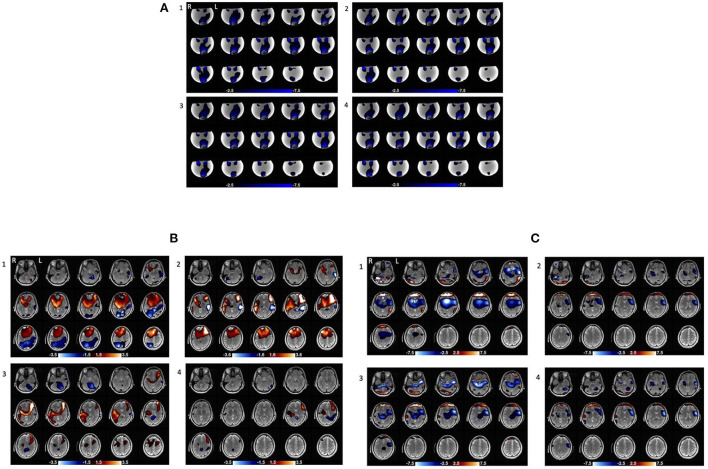
Current distribution maps in four consecutive stimulation scans (repeated with no change) in the phantom and one of human subjects. **(A)** Current distribution maps across the four consecutive stimulation scans in the phantom. **(B)** Magnetic field maps across the four consecutive stimulation scans in subject_10_. The areas with red and blue gradient represent positive and negative polarity, respectively. **(C)** Current distribution maps across the four consecutive stimulation scans in subject_10_. The blue area shows Right to Left current (J_x_) flow (the primary direction) and the red area shows Left to Right current (J_x_) flow which may be an artifact of boundary conditions at the edge of the brain near the frontal orbital region where susceptibility changes rapidly.

**Figure 6:**
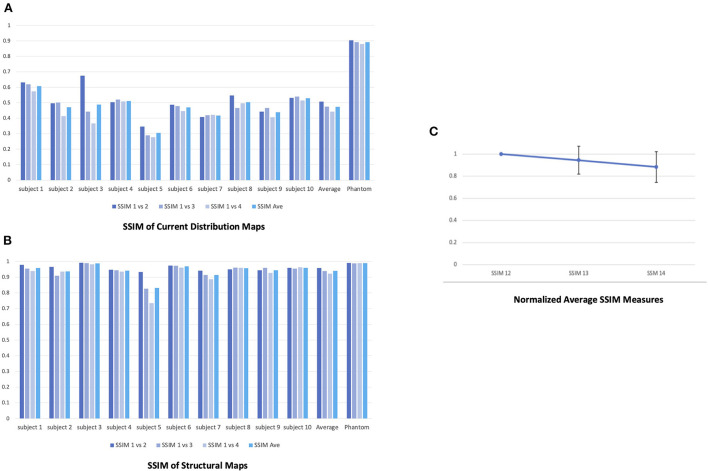
Repeatability of the four consecutive stimulation scans in the phantom and human subjects. **(A)** SSIM measures of the current distribution maps across the four scans in the phantom and human subjects. For both the phantom and each human subject, we took the current distribution map from the first of the four consecutive scans as reference and calculated SSIM between the maps from the reference and second (SSIM12), the reference and third (SSIM13), and the reference and fourth scans (SSIM14) to quantify the changes in the maps. **(B)** SSIM measures of the structural maps across the four scans in the phantom and human subjects. **(C)** The normalized average SSIM measures of the current distribution maps across all subjects. The normalized SSIM measures showed a decreasing trend in the similarity of the current maps across the time [1 (SSIM12); 0.945 (SSIM13; STD = 0.126); 0.884 (SSIM14; STD = 0.139)].

In addition, the result demonstrated significant intra-subject variability across four consecutive scans (shown for one subject, Figures 5B, C).^1^ The SSIM measure also showed lack of repeatability of the four current distribution maps in each subject; the average SSIM measure was 0.507 between the first and the second scan, 0.475 between the first and third scan, and 0.443 between the first and the fourth scan (average 0.474) (Figure 6A). The measures showed greater decreasing trend than those for the phantom experiment. However, the SSIM measures of the structural maps still showed high repeatability; 0.958 between the first and the second scan, 0.939 between the first and third scan, and 0.922 between the first and the fourth scan (average 0.940) (Figure 6B). The normalized average SSIM measures across the subjects also confirmed a temporal pattern of decrease in the similarity of the current maps across the repetitions of the scan (Figure 6C).

## 4. Discussion

In this study, we examined the tDCS-induced current distribution *in vivo* and in a phantom. By using a functional MRI data acquisition and analysis method, this study generated current distribution maps, although only one (*B*_*z*_) spatial component of the magnetic field was measured. Compared with previous studies (Jog et al., 2016, 2020, 2021; Kasinadhuni et al., 2017; Goksu et al., 2018, 2021), the crucial advantage of our approach is that it uses a block trial with slow periodic bipolar modulation of the current to increase the signal-to-noise ratio (SNR) and employs fMRI-like time-series analysis to reconstruct the magnetic field with high sensitivity. It also uses a waveform specifically designed to maximize the amount of RMS current delivered to the brain without exacerbating the unpleasant side effects of tDCS, such as tingling/itching by increasing the current duty cycle and changing polarity more smoothly. We indicated Right-Left current directions, although the actual stimulation current was slowly oscillating in both directions as shown in Figure 1B. By this we mean the GLM-derived proportionality of the measured (oscillating) magnetic field change and the oscillating stimulus current.

### 4.1. Primary outcomes

The results of the phantom experiment confirmed that our approach can reliably detect current distribution in the brain while undergoing tDCS. Conforming with the basic assumption of tDCS and previous modeling studies, the current distribution maps from the phantom showed a clear path of current flow between the two electrodes capturing more than 75% of the injected current, with a high repeatability across the four consecutive scans (average SSIM = 0.891). The phantom results also suggested that for the simple montage used in this study the wires did not appreciably distort the field maps.

However, the human subject experiment demonstrated that for the human brain, which comprises various types of tissue that have vastly different conductivities, the current was less evenly distributed in the sagittal plane (Figure 4B). In addition, only some portion of injected current [average 0.33 mA; 26.4% of the injected current (1.25 mA)] actually flowed through the cortex. This result suggests that a significant fraction of the current is bypassing the brain interior and traveling from one electrode to the other external to the brain through the highly conductive CSF and the scalp. In this study, we did not conduct a simulation modeling of current distribution. Previous *in-vivo* current mapping studies have already simulated a current path with a right-left montage identical to that of this study, reporting non-negligible differences between the simulated path and the measured path [e.g., about 30% (Kasinadhuni et al., 2017)] even though the missing components of the magnetic field were filled with the predictions from the modeling to generate the measured path (Kasinadhuni et al., 2017; Goksu et al., 2018).

The attenuation of current penetrated into the brain was reported in previous studies. For example, Voroslakos et al. (2018) showed that in rodents and human cadaver brains, the scalp, subcutaneous tissue, and muscles shunt about 50% of applied current intensity and that the skull will further reduce the current flow by another 10–25% depending on the skull's thickness, similar to what we found in the present study. They argued that a substantially larger amount of current than a conventional transcranial electrical stimulation protocol (1–2 mA) should be applied to attain a voltage gradient in brain tissue that is sufficient to affect neuronal firing.

Substantial inter-subject variability regarding the maximum total sum of current detected in the brain was also observed. The magnitude of Right (R) to Left (L) (J_x_) current averaged in 3-pixel-thick slabs in individual subjects (see Supplementary Figure 2) demonstrated much less constancy of current, or heterogeneous current path, across the brain, compared with magnitude of Posterior (P) to Anterior (A) (J_y_) current in 3-pixel-thick slabs in phantom (Figure 3B). It has been suggested that various factors, including but not limited to head size, tissue thickness, subcutaneous fat levels, CSF density, cortical fluid density, cortical surface topography, individual morphologies of cortical gyri and sulci, can influence the current path and result in significant inter-subject variability (Bikson et al., 2012; Datta et al., 2012; Opitz et al., 2015).

In addition, we also showed that the current distribution maps from individual human subjects were less replicable across the four consecutive stimulation scans compared with those from the phantom (Figures 5, 6). The average SSIM measure of the current maps in human subjects (0.474) was only about half of the measure in the phantom (0.891). Moreover, the high SSIM measures between structural maps in human subjects indicates that there was no overall structural image degradation across the scans and thus, the lack of repeatability in the current maps was primarily due to the changes in the current path. This significant intra-subject variability in the current maps suggests potential temporal evolution in the current distribution in the human brain as the stimulation repeated several times in the same montage. This evolution, which is indicated in the decreasing trend of the SSIM measures, could be resulted from changes in contact of the electrodes with the scalp through evaporation of electrolyte and the presence of hair. It may also be possible that the intra-subject variability in the current distribution maps is in part due to the current-induced hemodynamic effects in the brain. Previous studies have shown that transcranial electrical simulation can evoke transient changes in the cerebral blood flow and blood volume (Zheng et al., 2011; Arora et al., 2021; Arora and Dutta, 2022b), which could in turn affect the current path by altering the overall conductivity of the neurovascular tissue.

### 4.2. Limitations of the study

The major limitation of this study is that it only measured the *B*_z_ component of the induced magnetic field, which accounts for some of the missing current. Yet we declined to augment these measurements with models of the missing components to avoid biasing the results (Kasinadhuni et al., 2017; Goksu et al., 2018, 2021). By repeating the scans twice while the head is tilted from its normal position, a complete picture of the magnetic field distribution can be obtained (Hernandez-Garcia et al., 2019), which would allow full characterization of the current flow. However, this technique may be impractical for routine use due to discomfort of head positioning and increased potential for head motion.

It is also critical to note that the magnetic field generated by the injected current cannot be measured outside the phantom or the brain where MRI phase (and amplitude) information is not available. This discontinuity of the magnetic field across the edge of the phantom or the brain will distort the estimates of the current. Nevertheless, the combined effects of measuring *B*_*z*_-only magnetic field, and only inside the phantom or the brain resulted in much smaller errors in total current in the phantom experiment than in the human subject experiment, suggesting that these intrinsic limitations cannot fully explain the low current observed in human subjects. Rather, as discussed above, the substantial reduction of the current in human subjects would probably be due to the deviation of the current to the CSF or the scalp where resulting magnetic field could also not be measured. Future studies can surround the head with a water-filled bag so that the magnetic field can be estimated outside the head for more complete calculation of current flow maps.

An additional limitation is the modest sample size (*N* = 10). However, the goal of this study is not to study neurocognitive effects of tDCS but to examine the injected current distribution in the brain, in an effort to explain the surprising lack of robustness in tDCS' neuromodulation in even a very simple montage with standard electrodes. Previous in-vivo current mapping studies also had comparable sample size [13 in Jog et al. (2016); 7 and 8 in Jog et al. (2020); 4 in Kasinadhuni et al. (2017); 13 in Goksu et al. (2018); 8 in Goksu et al. (2021)]. The age range (23–79) can also be considered quite large given the sample size, but there was no statistically significant relationship between subjects' age and the maximum total sum of current in the average current map across the four scans (Supplementary Figure 3).

This study used the conventional size of saline-soaked sponges (5 × 7 cm^2^) to reduce the unpleasant tingling or itching sensation, but they still cover a large area and could contribute to imprecise targeting. We also wrung the sponges out to remove extra saline, but it is possible that remaining saline permeates nearby areas through hair or elastic bandages used to affix the sponges and cause variability in the current maps across the four stimulation scans. Future studies can further improve the measurement of the tDCS-induced magnetic field and current distribution by using smaller electrodes [e.g., HD tDCS (Parlikar et al., 2021)]—to the extent that it does not heighten safety risks by increasing the local current density under the electrodes.

Finally, we did not measure the hemodynamic changes caused by the injected current in this study. However, given the potential cerebrovascular reactivity to the current reported in previous studies (Zheng et al., 2011; Arora et al., 2021; Arora and Dutta, 2022a), further studies can examine the correlation between the current path and the cerebral blood flow/ blood volume changes which may in part explain the intra-subject variability across the four scans.

## 5. Conclusion

tDCS is a non-invasive neuromodulation technique that has been widely utilized both as a therapy for various neuropsychiatric diseases and for cognitive enhancement for healthy subjects. Yet recent reports on inconsistent outcomes of tDCS have called for a more rigorous investigation of the underlying mechanism of tDCS. Our study proposes a functional MRI technique to measure the tDCS-induced current distribution in the brain. Despite the limitation that this technique can only detect partial current flow, our results in an experiment that minimized the impact of this limitation confirmed that much of the injected current was not accounted for in human brain relative to that in the phantom (only 1/3), suggesting that much of the missing current was flowing at the edge of the brain where it was not measured. We also found substantial inter and intra subject variability in current distribution maps. The lack of repeatability in the current distribution maps across the four consecutive scans suggests the potential temporal evolution in the current distribution in the human brain resulting from changes in contact of the electrodes with the scalp. These findings would have ramifications in the use of tDCS as a neuromodulator and may help explain some of the inconsistencies reported in other studies.

## Data availability statement

The raw data supporting the conclusions of this article will be made available by the authors, without undue reservation.

## Ethics statement

The studies involving human participants were reviewed and approved by Stanford University Institutional Review Board. The patients/participants provided their written informed consent to participate in this study.

## Author contributions

AJ: conceptualization, formal analysis, investigation, methodology, writing—original draft, and writing—review and editing. GG: conceptualization, formal analysis, investigation, methodology, supervision, funding acquisition, and writing—review and editing. JG: formal analysis, methodology, and writing—review and editing. All authors contributed to the article and approved the submitted version.

## Footnotes

^1^This subject's current maps in four consecutive scans show a similarity between scan 1 and scan 3 and between scan 2 and scan 4. However, this was an incidental pattern and did not appear in other subjects' current maps.

## Supplementary material

Supplementary Figure 1 **(A)** B maps in NaCl-doped water phantom at currents of 1.25–0 mA, from beta maps of GLM. Electrodes are at top and bottom of phantom, thus current flow should be vertical, but asymmetry in electrode placement causes skew. Red portrays induced B field out of the image plane; blue is opposite direction; both are parallel to main magnetic field. **(B)** ROI in positive (red) magnetic field plotted for the 6 measured maps, with linear fit overlaid. Departure from linearity demonstrates noise limit.

Supplementary Figure 2 Average current distribution map across four consecutive scans in ten subjects and magnitude of right (R) to left (L) current flow (J_x_) in 3-pixel-thick slabs in the sagittal plane. The blue area shows Right to Left current (J_x_) flow (the primary direction) and the red area shows Left to Right current (J_x_) flow which may be an artifact of boundary conditions at the edge of the brain near the frontal orbital region where susceptibility changes rapidly.

Supplementary Figure 3 The effect of age on maximum total sum of current in ten subjects. No statistically significant relationship between age and maximum total sum of current was observed (Pearson's *r* = −0.105).
