# Supplementary figures and images for: Inconsistencies in mapping current distribution in transcranial direct current stimulation
Source: Front Neuroimaging. 2023 Jan 16;1:1069500. doi: 10.3389/fnimg.2022.1069500 (PMC10406311; doi:10.3389/fnimg.2022.1069500)

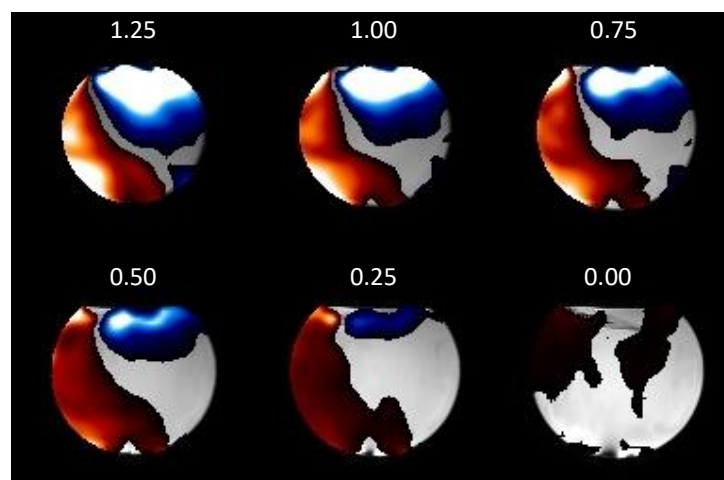

(A)

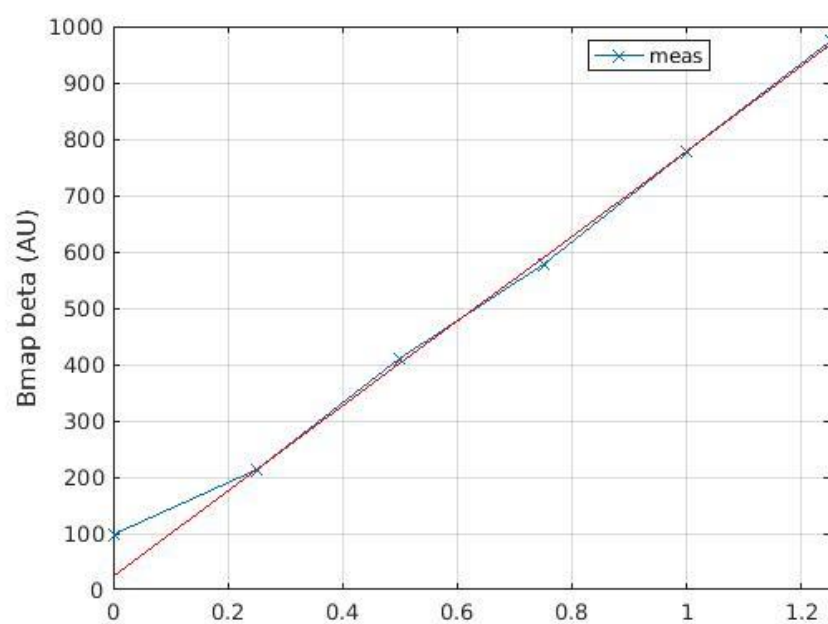

(B)

Supplement: Supplementary Figure 1 — (A) B maps in NaCl-doped water phantom at currents of 1.25–0 mA, from beta maps of GLM. Electrodes are at top and bottom of phantom, thus current flow should be vertical, but asymmetry in electrode placement causes skew. Red portrays induced B field out of the image plane; blue is opposite direction; both are parallel to main magnetic field. (B) ROI in positive (red) magnetic field plotted for the 6 measured maps, with linear fit overlaid. Departure from linearity demonstrates noise limit. [file Image_1.pdf]

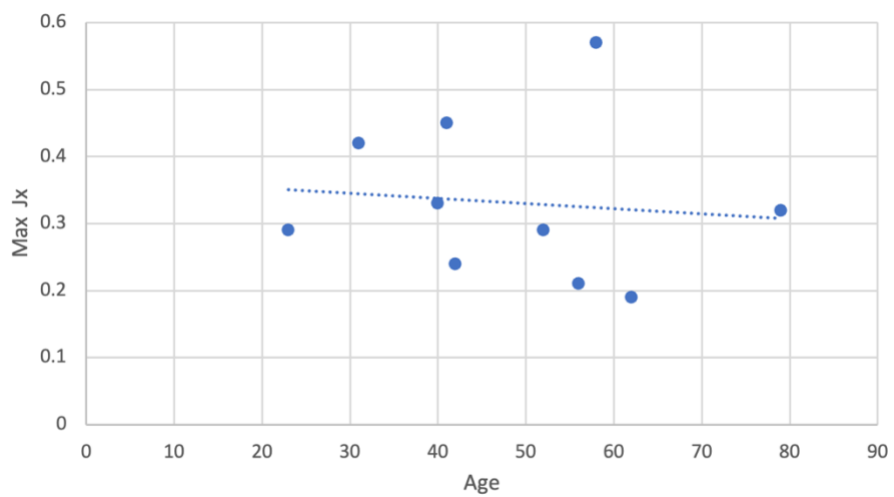

Supplement: Supplementary Figure 3 — The effect of age on maximum total sum of current in ten subjects. No statistically significant relationship between age and maximum total sum of current was observed (Pearson's r = −0.105). [file Image_3.pdf]
