# Supplementary material for: Inconsistencies in mapping current distribution in transcranial direct current stimulation
Source: Front Neuroimaging. 2023 Jan 16;1:1069500. doi: 10.3389/fnimg.2022.1069500 (PMC10406311; doi:10.3389/fnimg.2022.1069500)
Supplement: Supplementary Figure 2 — Average current distribution map across four consecutive scans in ten subjects and magnitude of right (R) to left (L) current flow (Jx) in 3-pixel-thick slabs in the sagittal plane. The blue area shows Right to Left current (Jx) flow (the primary direction) and the red area shows Left to Right current (Jx) flow which may be an artifact of boundary conditions at the edge of the brain near the frontal orbital region where susceptibility changes rapidly. [file Image_2.pdf]

Subject $_1$

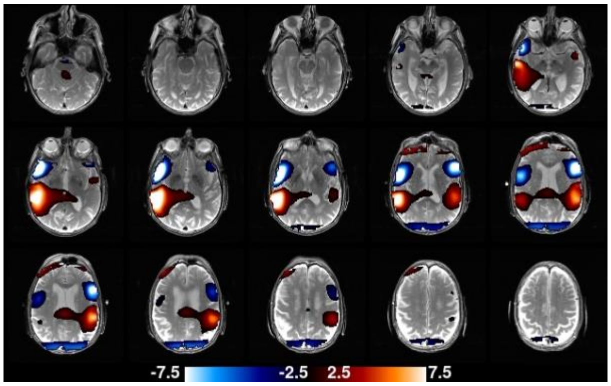

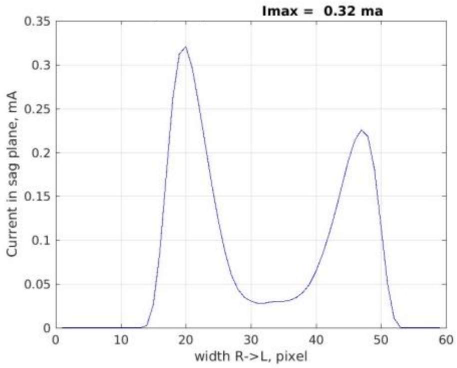

Subject $_2$

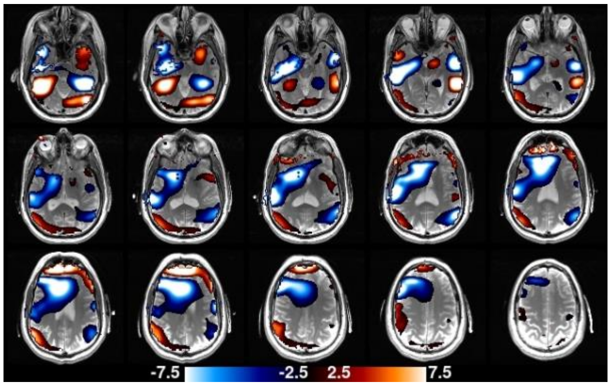

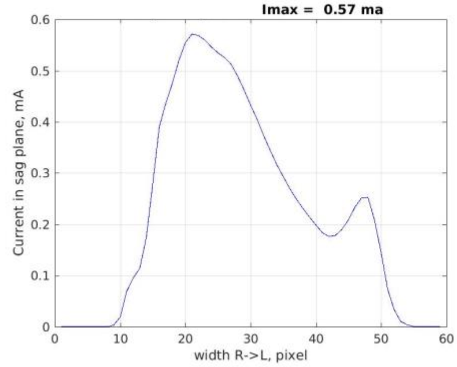

Subject $_3$

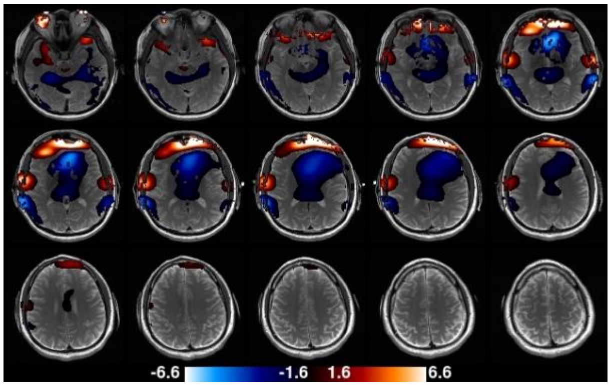

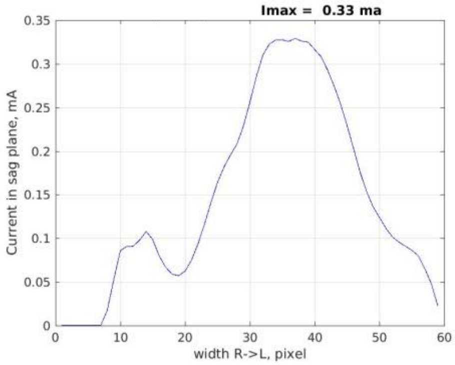

Subject $_4$

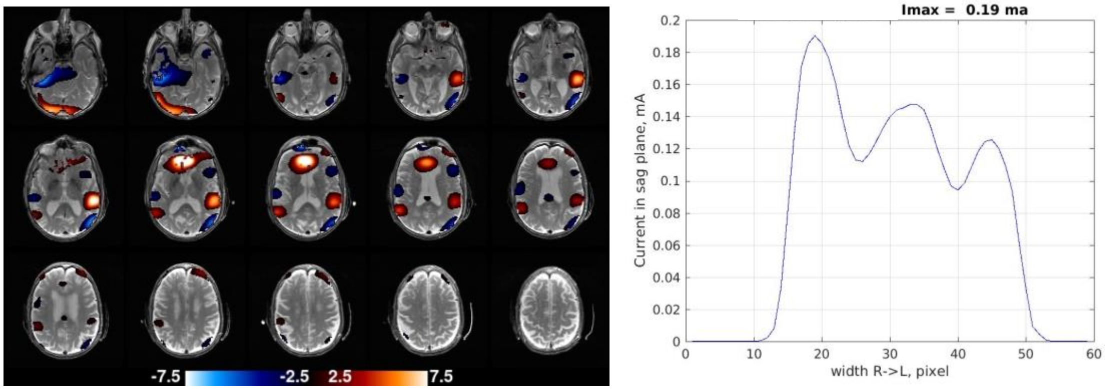

Subject $_5$

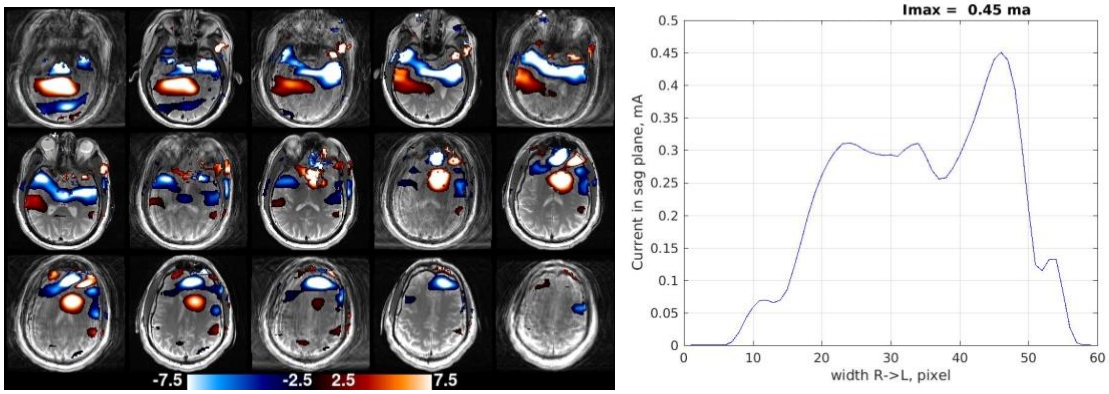

Subject $_6$

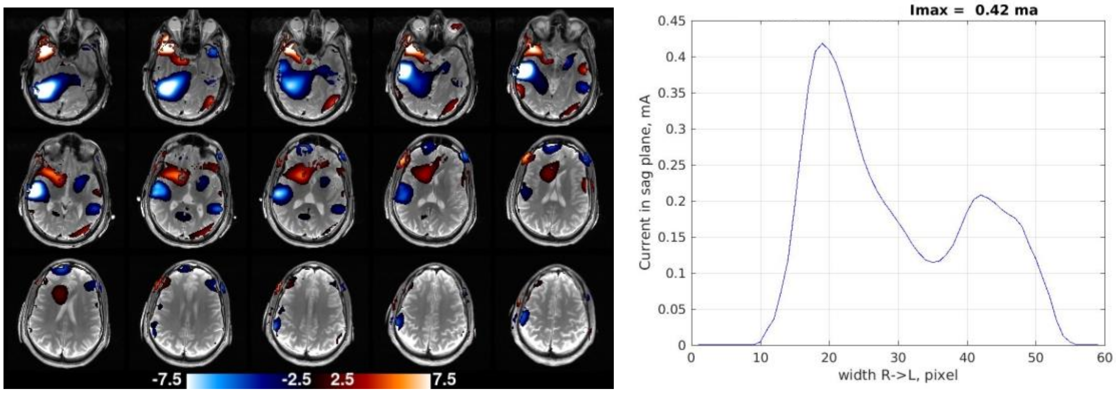

Subject 7

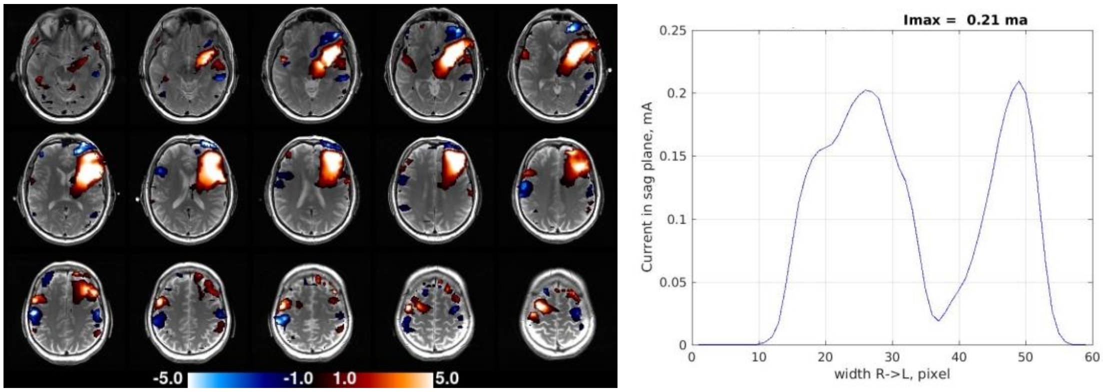

Subject 8

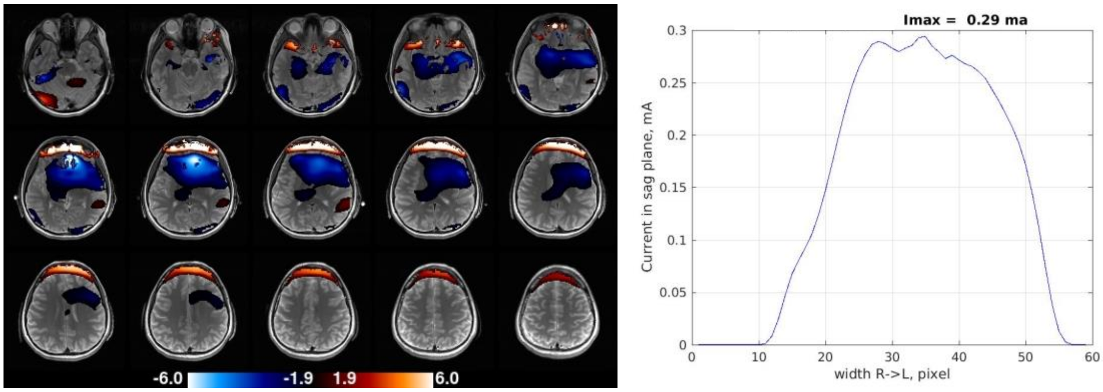

Subject 9

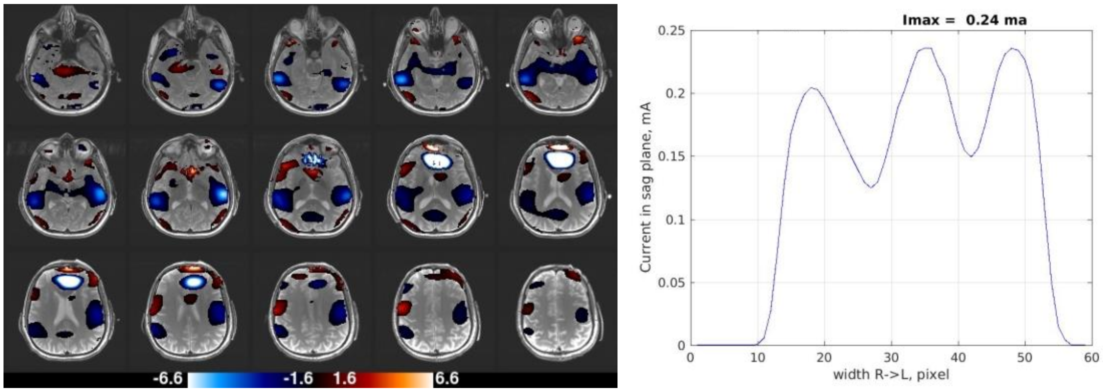

Subject $_{10}$

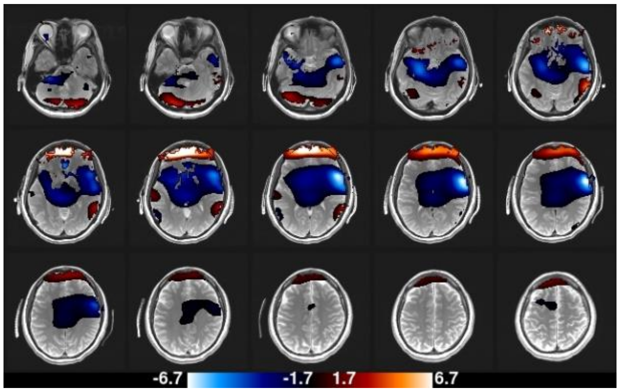
-6.7
-1.7
1.7
6.7

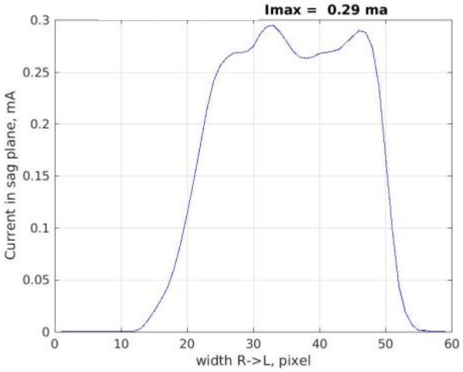
Imax = 0.29 ma
Current in sag plane, mA
width R->L, pixel
0
0.05
0.1
0.15
0.2
0.25
0.3
0
10
20
30
40
50
60
